# The Mystery behind the Pineal Gland: Melatonin Affects the Metabolism of Cholesterol

**DOI:** 10.1155/2019/4531865

**Published:** 2019-07-10

**Authors:** Kamil Karolczak, Cezary Watala

**Affiliations:** Department of Haemostatic Disorders, Chair of Biomedical Sciences, Medical University, ul. Mazowiecka 6/8 92-215 Lodz, Poland

## Abstract

Melatonin may be considered a cardioprotective agent. Since atherogenesis is partly associated with the metabolism of lipoproteins, it seems plausible that melatonin affects cardiovascular risk by modulating the metabolism of cholesterol and its subfractions. Moreover, cholesterol-driven atherogenesis can be hypothetically reduced by melatonin, mainly due to the minimalization of harmful reactions triggered in the cardiovascular system by the reactive oxygen species-induced toxic derivatives of cholesterol. In this review, we attempted to summarize the available data on the hypolipemizing effects of melatonin, with some emphasis on the molecular mechanisms underlying these reactions. We aimed to attract readers' attention to the numerous gaps of knowledge present in the reviewed field and the essential irrelevance between the findings originating from different sources: clinical observations and *in vitro* mechanistic and molecular studies, as well as preclinical experiments involving animal models. Overall, such inconsistencies make it currently impossible to give a reliable opinion on the action of melatonin on the metabolism of lipoproteins.

## 1. Introduction

In this review, we analyzed the results from clinical, animal, and *in vitro* studies investigating the role of melatonin in the regulation of metabolism of cholesterol, along with data on the capability of melatonin to reduce the cardiovascular toxicity of lipids and their harmful derivatives. We made an attempt to identify gaps of knowledge in experimental approaches in order to conclude how, if at all, melatonin affects the metabolism of cholesterol.

## 2. Melatonin as a Beneficial Regulator of Cholesterol Metabolism: A Phenomenon Observed at the Laboratory Level Relevant to Clinical Observations with Not Entirely Identified Molecular Basis

Animal models provide evidence of the beneficial regulation of cholesterol by melatonin. Rats with diet-induced hypercholesterolaemia that are treated orally with melatonin at a dose of 10 mg/kg/day exhibited significantly improved blood plasma cholesterol profiles. Subsequently, these profiles were characteristically antiatherogenic: total cholesterol, very low-density lipoprotein (VLDL), and low-density lipoprotein (LDL) cholesterol concentrations decreased, whereas high-density lipoprotein (HDL) cholesterol concentrations increased [1]. Another study yielded similar and proportional results to that of Hussain, and melatonin-induced changes to cholesterol were observed not only in rats with high-cholesterol diet-triggered hypercholesterolaemia,but also in models of hypercholesterolaemia associated with hypothyroidism in rats [2].

Rats treated with increasing doses of lopinavir and ritonavir exhibited higher concentrations of total cholesterol, LDL, VLDL, and HDL cholesterol. All these increases in cholesterol were significantly reduced by melatonin; however, the highest degree of eucholesterolaemic action of melatonin was observed when it was used together with lipoic acid [3]. Similarly, melatonin can inhibit health-threatening shifts in lipoprotein profile induced by other drugs, like methadone and antipsychotics, or toxic compounds, including aluminium [4–6].

This implies that melatonin is an efficient cholesterol stabilizer regardless of the cause of hypercholesterolaemia (diet, metabolic disorders associated with hormonal impairment, side effects of pharmacotherapy, etc.). Such implications, however, should be verified with further studies on lipid diseases, filling gaps with information that is currently unavailable.

All above data encourage to consider melatonin as a very promising and quite universal hypolipemizing agent (regardless of the cause of dyslipidaemia). Unfortunately, such promising reports are usually occasional, and thus, further experimental confirmations are urgently needed and even more so, considering the fact that different experimental approaches may lead to inconsistent conclusions, as we attempt to evidence and emphasize below in the text.

In animal models of type 2 diabetes—like rats with streptozotocin-induced diabetes fed with a high-fat diet—characterized by hypercholesterolaemia, melatonin failed to significantly decrease the levels of total cholesterol and LDL cholesterol. This model of diabetes, as induced by the concomitant hyperglycaemia and high-fat diet, was characterized by decreased nitric oxide concentrations and impaired endothelium-dependent vasorelaxation. Interestingly, all these impairments are, at least partially, dependent on a higher ratio of LDL/HDL [7–12]. Moreover, all these disorders are effectively improved by melatonin, which increases NO concentration, and improves endothelium-dependent vasorelaxation. Since lipoproteins were not affected by melatonin in models with high-fat diets and streptozotocin-induced type 2 diabetes, all the aforementioned melatonin-dependent improvements are seemingly unrelated to melatonin lipid regulation; this suggests, instead, that cells comprising the vascular walls are the primary target for melatonin in terms of vasoregulation. However, further explication of this hypothesis is now beyond the scope of the present review.

It should be emphasized that after melatonin treatment, the values of lipoprotein cholesterol concentrations were always lower than in the control group (streptozotocin-induced diabetes+high-fat diet+no melatonin supplementation); however, these lower values were not statistically significant. It raises the question of whether melatonin introduced at higher doses than those employed (10 mg/kg/day) would bring more distinctive changes in animals with the mixed model of cholesterolaemia+streptozotocin diabetes [13]. For example, the dose of melatonin equal to 15 mg/day/kg b.w. was tested in rats with streptozotocin diabetes for 15 days and was characterized as a very effective reducer of diabetes-induced increases in total cholesterol levels [14]. However, the final conclusions derived from the study of Salmanoglu et al. [13] and another one of Akmali et al. [14] should be compared and interpreted with great caution, because the first team worked with a streptozotocin diabetes paralleled by hypercholesterolaemia, whereas the second team worked with a streptozotocin diabetes not “burdened” with other pathophysiological risk factors. However, not only the model of metabolic pathology,but also the way the hormone is administered can be essential. Diverse routes of melatonin administration may certainly matter and are worth considering, as they could result in significantly different effects. Noteworthily, the routes of treatments with investigated drugs can dramatically alter the final outcomes in streptozotocin-induced diabetic rats, at least in terms of glycaemic control [15]. Moreover, a time point of melatonin therapy (started before or after the induction of diabetes with streptozotocin) can also significantly affect its action in this particular model of hyperglycaemia [14, 16]. Interestingly, in another model of diabetes, i.e., in Zucker diabetic rats, melatonin at the same daily dose as used in the study by Feron et al. [11] reduced LDL, raised HDL, and did not affect total cholesterol [17].

Few studies have previously investigated the issue of variations in effectiveness caused by different routes of melatonin administration. Outcomes from transdermal, oral, subcutaneous, intranasal, and intravenous routes of melatonin administration are not equal [18, 19]. Thus, the effectiveness of cholesterol control by exogenously introduced melatonin can vary widely, producing different results depending on the route of melatonin administration, especially when the tested animal model includes very complex metabolic disorder, like diabetes.

Comparing the studies of Hussain [1], Chan and Tang [2], and Salmanoglu et al. [13], it can be speculated that the beneficial effects of melatonin on a lipid profile are hampered by concomitant diabetes and possibly by any other coexisting metabolic disorders. Thus, melatonin appears to be an effective cholesterol stabilizer in pure models of hypercholesterolaemia. In mixed models of metabolic diseases, melatonin may require support from other molecules, like lipoic acid [3].

An inverse correlation between melatonin concentration and HDL levels in patients with metabolic syndrome did not give a solid foundation to raise the hypothesis that the increase in melatonin concentration will increase HDL levels and decrease the levels of LDL and total cholesterol [20]. Also, other observations demonstrated that in the elderly (people aged 73 yr on average), the overnight exposure to the illumination lighter than 3 lux is associated with higher risk of obesity and higher levels of total and LDL cholesterol concentrations, despite the fact that the urine melatonin concentrations remained without any relevance to these associations [21]. These findings seem to apparently discourage the hypothesis on any straightforward involvement of melatonin in the shaping of cholesterol metabolism.

Nevertheless, some other studies estimating the role of melatonin supplementation in the treatment of lipid disorders that have been performed even earlier in some particular groups of human participants remain even more inconclusive. In the trial with human participants, melatonin was administered at the dose of 6 mg/day for two weeks, but it did not result in the altered concentrations of total cholesterol, LDL, or HDL cholesterol in normolipidemic postmenopausal women [22]. The resultant conclusion was that melatonin supplementation did not significantly change the levels of HDL or LDL lipoproteins. However, melatonin increased concentrations of VLDL cholesterol subfraction [22]. Very similarly, melatonin (0.3 mg/day or 3 mg/day for 6 weeks, taken at bedtime) used in patients with hypercholesterolaemia did not affect plasma lipidogram parameters. However, some trend towards lower concentrations of total cholesterol and LDL cholesterol occurred in subjects who received the higher dose of melatonin [23]. Quite similarly, among the women (40-60 years old), those who were given melatonin for 3 months at the dose of 3 mg daily did not evoke any changes in lipid parameters [24]. On the contrary, melatonin administered at considerably higher doses (10 mg daily for 3 months) decreased total and LDL cholesterol levels, probably due to the upregulation of the expression of LDL receptor, in women with polycystic ovary syndrome [25]. Also, in diabetic patients with coronary artery disease, a higher regimen of melatonin dosing (10 mg daily for 12 weeks) improved HDL and total/HDL cholesterol ratio [26].

The above brief reviewing of the outcomes originating from several clinical studies dealing with hypolipemizing effects of melatonin definitely drives to a quite certain conclusion that any significant effects of melatonin on plasma lipoproteins were revealed merely at the highest tested melatonin concentrations (10 mg daily), while the lower doses remained ineffective. Overall however, the relation between melatonin dosing and its effectiveness still remains elusive, particularly when we address the question of what is the lowest melatonin dose effective in remarkable lipoprotein changes to another well-designed clinical study by Goyal et al. [27]. In a double-blind, placebo-controlled, crossover pilot clinical study, a higher dose of melatonin (8 mg/day) and a longer treatment time (10 weeks) were employed. The study revealed only moderate and insignificant effects of melatonin on cholesterol fractions [27]. Thus, the notion that higher doses of melatonin used for longer periods of treatment lead to a significant reduction in total cholesterol and triglyceride levels, as shown in the meta-analysis of controlled randomized trials, is not always supported [28].

Two major issues, referring to the problem of a dose, should be, however, emphasized herein. Again, the comparing of the outcomes coming from all these diverse studies should be a matter of great awareness, since they were performed with different study populations (postmenopausal women, subjects with polycystic ovary syndrome and diabetic patients with coronary artery disease). More importantly, much larger well-designed clinical studies, with a reliably estimated required minimum sample size and a desirable statistical power, are needed in order to obtain more reliable and validated data. Regrettably, a very interesting and promising study by Raygan et al. [26] has been made on 30 subjects, which defines this work rather as a pilot study. In spite of that, however, it brings a very promising material encouraging to undertake much larger observations.

With reference to what was written above, we have to be aware that the problem of dosing is only just one face of the coin and should not be discussed in separation from other issues, like clinical and demographical background of studied populations. In the aforementioned study from Goyal et al., the hypothesized changes in HDLs and LDLs after melatonin treatment were investigated as components of metabolic syndrome; thus, the effects of melatonin may have been weakened by concomitant metabolic disorders similar to the experiments concerning the effects observed in the course of melatonin eucholesterolaemic therapy in mixed animal models [27]. Importantly, the authors assessing the *in vivo* effects of melatonin in humans often declare themselves that the selection of the dose of melatonin used in a given study is quite arbitrary, simply because it is difficult to predict which melatonin concentration is appropriate to induce the desired effect in a given group of patients [29]. This makes any standardization of the observed effects to a cumulative melatonin dose ever more difficult, if possible at all.

At this point, the question may be raised of whether we are able to track the molecular pathway(s) underlying a potential impact of melatonin on the key steps of lipid metabolism, like the absorption of cholesterol from diet, different stages of cholesterol synthesis, activities of the pivotal cholesterol-metabolizing enzymes, e.g., lipoprotein lipase and cholesterol acyltransferase or the expressions of LDL receptors.

It was suggested that cholesterol from one's diet is absorbed in the intestine by cholesterol-transporting proteins Abcg5 and Abcg8, but this hypothesis was not supported by experimental findings [30]. Further, decreased expression of Abcg5 and Abcg8 in the colon may lead to increased cholesterol absorption, at least under diabetic conditions [31]. Thus, until the controversial mechanism of cholesterol absorption is not clarified, it is difficult to speculate on the molecular background underlying the melatonin-induced increase in absorption of diet-derived cholesterol [1]. Nevertheless, another hypothesis was raised pointing to the possible melatonin-mediated epigenetic regulation of Abcg protein expression; this hypothesis suggests that regulation occurs via altering the methylation status of the gene(s) coding Abcg proteins [32].

The liver is the second most important place of cholesterol metabolism. Hepatic synthesis of bile acids is a key step in cholesterol excretion from the liver and biliary secretion of free cholesterol [33], which is regulated by melatonin [2].

Looking at the process of cholesterol metabolism and its sensitivity to melatonin, we can learn, for example, that melatonin is able to reduce the synthesis of cholesterol in mononuclear leukocytes, but it does not inhibit the synthesis of lanosterol. Thus, it appears that melatonin interferes with the synthesis of cholesterol somewhere in the middle of the pathway, probably not before the synthesis of the first cyclic precursor in the *de novo* synthesis of sterols [34]. It can be concluded that melatonin's effect on the different steps of cholesterol metabolism should result in decreased levels of extra- and intracellular cholesterol. Surprisingly, however, the results showed that despite the melatonin-mediated inhibition of cholesterol synthesis, the expression of LDL receptors, a crucial target in the process of extracellular cholesterol absorption, decreased [34]. It differs and opposes the action of melatonin from that typically revealed for statins in hepatocytes [35]. The binding affinity of LDL molecules to LDL receptors is not affected by melatonin [34].

It is probable that due to its interference with cholesterol metabolism at different stages of cellular metabolism, melatonin can reduce the total quantity of cholesterol in some organs, like the liver or brain [36]; however, the observed accumulation of lipids in fibroblasts suggests that melatonin stimulates lipid storage in a process sensitive to luzindole, a selective melatonin receptor antagonist [37, 38]. Specific cell and tissue differences come to mind first when attempting to clarify this discordance, but further studies are needed to test this complex matter to a considerable extent. It seems obvious that in order to prove how complex and fascinating melatonin's potential contribution is to lipid metabolism, it is necessary to first acquire results showing the inhibition of lipoprotein lipase by melatonin [22]. Lipoprotein lipase is the primary controller of lipid metabolism, with a distinct impact on tissue-specific patterns of lipid accumulation, insulin resistance, glucose utilization, and obesity [39]. Wakatsuki et al. were the first researchers to provide evidence of the sensitivity of lipoprotein lipase to melatonin [22]; however, more detailed studies with regard to tissue-specific aspects of melatonin's effects on lipoprotein lipase activity (muscles versus adipocytes versus cardiomyocytes, for example) should be urgently undertaken/ventured.

What is the molecular evidence which can be found at present to support the hypothesis that melatonin affects metabolism of cholesterol? Recently published results of experiments performed on HepG2 cells incubated with oleic acid and treated with melatonin have shown that the expression of 3-hydroxy-3-methylglutaryl CoA reductase (HMGCoA-R) and sterol regulatory element-binding protein 2 (SREBP-2) remained unchanged in the presence of melatonin. However, melatonin increased the expression of peroxisome proliferator-activated receptor-*α* (PPAR*α*) and its target gene, carnitine palmitoyl-CoA transferase 1 (CPT1) [40]. In turn, the latter changes may be involved in the modulation of lipoprotein levels. The activation of PPAR*α* may lead to the increased plasma HDL concentration and the lowered concentration of LDL, probably in the manner dependent on the increased expression of LDL receptor in liver cells [41, 42]. It is because the increased expression of PPAR*α*, caused by melatonin, may facilitate the activation of the LDL receptor. Thus, it is possible that the supplementation with melatonin may plausibly enhance the effectiveness of the lipid-lowering action of PPAR*α*-dependent agonists.

According to our humble opinion, the most prominent evidence showing the significant role of melatonin in cholesterol metabolism has been provided by the experiments done with animals or even human subjects subjected to pinealectomy. Pinealectomized animals have been shown to be characterized by raised cholesterol levels [43, 44], similar to human subjects who underwent pinealectomy or experienced melatonin deficiency due to other reasons, like radiotherapy [45]. Interestingly, in both animal and human studies involving pinealectomy, the melatonin replacement therapy reversed the atherogenic profile of lipoprotein subfractions [43–45]. Needless to emphasize, very valuable studies using pinealectomized animals have been undertaken only occasionally, their outcomes are very few, and certainly, they should be performed more often to further validate the role of melatonin in regulation of lipid metabolism.

## 3. Melatonin Protects Cells against Toxic Action of Oxidized Lipoproteins

Patients with acute myocardial infarction are characterized by lower levels of nocturnal melatonin and higher levels of oxidized low-density lipoproteins, which manifest harmful effects on the cardiovascular system. In a model system, transient metal ions are required for the oxidation of LDL particles. Melatonin has been shown to decrease the oxidative derivatization of apolipoprotein B induced by copper ions, but despite the prolonged lag time of conjugated diene formation [46, 47], melatonin did not reduce the final diene quantities under *in vivo* conditions [46]. It seems that melatonin effectively decreases the quantity of dienes only under *in vitro* conditions and especially at nonphysiologically high concentrations [47]. It is still unclear, however, whether melatonin equally protects LDLs from oxidation in its lipid and protein domains. Some authors suggest that melatonin may effectively protect only polyunsaturated acids of LDL against the attack of reactive oxygen species. Furthermore, the protein core of lipoproteins may be less effectively protected and can be transformed by copper-induced ROS into atherogenic derivatives, even in the presence of melatonin. One very interesting idea suggests that some derivatives of melatonin might form adducts with apolipoprotein B, thus leading to its oxidative transformation [48].

The true antioxidant properties of melatonin are questionable, primarily because the *in vitro* conditions of these experiments are different from physiological “reality.” First, in numerous studies, supraphysiological concentrations of melatonin have been used. Further, when compared to others, melatonin is a relatively weak antioxidant; it is much weaker than tocopherol, ascorbic acid, or tryptophan [47, 49, 50]. In regard to the formation of conjugated dienes, melatonin appears to serve as a weaker antioxidant than its own derivatives—GWC20 ((R,S)-1-(3-methoxyphenyl)-2-propyl-1,2,3,4-tetrahydro-beta-carboline) and DTBHB (N-[2-(5-methoxy-1H-indol-3-yl)ethyl]-3,5-di-tert-butyl-4-hydroxybenzamide) [51]. Thus, the results suggest that a strong melatonin-dependent antiatherogenic action occurs due to a robust reduction of LDL oxidation. Hence, inhibition of all the toxic effects triggered by oxidized LDLs should be considered with caution.

Melatonin's ability to inhibit transient metal ion-mediated lipoprotein oxidation should be cautiously reevaluated, especially considering the poor translation of *in vitro* experiments to *in vivo* environments, where metal ions are generally trapped and become incapable of providing redox reactions in plasma or specific areas of the body, like the interstitial fluid in an arterial wall. Generally, body fluids in different parts of an organism are well protected against extensive oxidation, which makes the putative contribution of melatonin to antioxidative status more elusive [52, 53]. Further research should be conducted on cellular systems that show increased lipoprotein oxidation potential rather than focussing on extracellular fluids. Among various blood cells, monocytes and macrophages are equipped with a few enzymatic systems that are potentially able to oxidize LDL, including ceruloplasmin, 15-lipoxygenase, and myeloperoxidase [54]. Ceruloplasmin, 15-lipoxygenase, and myeloperoxidase have already been shown to serve as LDL oxidizing agents [55–58]. To the best of our knowledge, neither of the aforementioned cell-derived enzymatic systems catalysing LDL oxidation in a transient metal ion-dependent manner has been shown to be sensitive to the antioxidant action of physiological (and even nonphysiological) concentrations of melatonin. Besides, monocytes, macrophages, and potentially other types of cells, including the endothelium, smooth muscle cells, and neutrophils, have the potential to oxidize LDL [59, 60]. Numerous cell types express melatonin receptors or are sensitive to the actions of at least some of the melatonin receptor antagonists [61, 62]. In the case of blood vessels, which are equipped with melatonin receptors [63], smooth muscle cells, but not the endothelium, are thought to be the primary site of melatonin receptors [61, 64]. In the case of receptor-dependent effects of melatonin on cholesterol metabolism, it would be very valuable, from a pharmacological point of view, to compare melatonin with other agonists of melatonin receptor, since some of them, for instance, piromelatine, are also able to raise the concentration of HDLs [65]. Even a lack of melatonin membrane receptors in some cells or an insensitivity to melatonin receptor antagonists does not rule out the modulatory effects of melatonin, since melatonin has also been demonstrated to act in a membrane receptor-independent manner [66]. Additionally, melatonin has been shown to act through other target proteins, like retinoic acid receptors and related orphan receptors/retinoid Z receptors [67], or via direct binding to cytosolic proteins, like calmodulin [68].

## 4. Melatonin Heals Cholesterol-Induced Cellular Impairments of Cell Membranes

Assuming that melatonin can indeed hamper the toxic effects of oxidized lipoproteins, phenomena that occur on or within cellular membranes deserve special attention. At increased concentrations, LDL cholesterol exerts a plethora of toxic effects that contribute to the atherogenic burden experienced by different tissues. The common phenomenon in numerous cells is the reduced membrane fluidity, induced by the accumulation of cholesterol within a membrane lipid bilayer (cholesterol may interact with membranes either as a single molecule [69] or as a component of lipoproteins [70]); this effect can be observed in both model membranes [69] and in a variety of different cells involved in cardiovascular homeostasis [70]. Decreased membrane fluidity purportedly triggers processes that perpetuate atherosclerosis, such as the adhesion of monocytes to the endothelial layer [70, 71], the first step in the development of atherosclerosis. Melatonin has been demonstrated to interact with lipid membranes and to fluidize them [72–75]. It is believed that melatonin localizes in the interfacial part of membranes and interacts through hydrogen bonding with phosphate groups of the polar heads of membrane phospholipids [76, 77]. A few particular features of melatonin-treated membranes serve as evidence of the fluidizing effect of melatonin, including increased membrane thickness [73, 74], induction of the gel phase, as a preferred state of a membrane lipid bilayer [73], and reduction of enthalpy in the lipid nonpolar chains or disordered lipid hydrocarbon chains [73, 74]. In regard to the interactions of cells with cholesterol, which has a rigidizing impact on cell membranes, melatonin can act as an efficient counteractor of cholesterol-induced membrane rigidity [75].

Melatonin's effects on the orderliness, or lack thereof, of the membrane lipid bilayer depend upon melatonin concentration and have been reported for both low and high melatonin levels, as shown in anionic dipalmitoyl phosphatidylglycerol (DPPG) multilamellar liposomes [77]. The presence of melatonin in distearoyl phosphatidylcholine (DSPC) model membranes has been demonstrated to reduce the temperature of the main phase, while melatonin treatment increased lipid dynamics in both the gel and liquid crystalline phases [78].

Moreover, it should be noted that melatonin is capable of passively moving across membranes [72] in addition to moving via facilitated transport through glucose GLUT transporters [79], which suggests that cytoplasmic proteins are accessible to melatonin and its effects.

## 5. Conclusions

Melatonin acts on cholesterol-dependent atherogenesis in a multifaceted manner. It can directly regulate the concentration of cholesterol in ways that are not well understood, and it can also rescue tissues from the harmful effects of oxidized lipoproteins. Thus, melatonin influences the “cholesterol branch” of atherosclerosis by influencing both the causes and the effects of hypercholesterolaemia/dyslipidaemia.

A wide range of experimental approaches have been used to define the impact of melatonin on cholesterol metabolism. Many of these approaches estimated serum concentrations of cholesterol subfractions, usually after a quantity of time that was too short for melatonin supplementation. In general, these experiments show that melatonin affects cholesterol levels rather insignificantly, but some studies have provided evidence for the significant and beneficial effects of melatonin. Well-planned controlled trials focused on the effects of long-lasting melatonin supplementation, with careful standardization for confounding factors such as traditional and nontraditional cardiovascular risk factors, age, and sex, are necessary in shedding more novel and unambiguous light on this field.

Our analysis of existing melatonin research showed that there is a lack of eucholesterolaemic effects, which was especially apparent in models with combined hypercholesterolaemia and some additional metabolic diseases, like diabetes.

We still do not have a comprehensive understanding of the molecular modulation of enzymatic processes (on the level of gene transcription and protein expression) involved in melatonin's effects on cholesterol metabolism. Thus, to date, the accomplished but quite novel results of this study should open a new era of investigations using contemporary methods of molecular biology to lead us to verification of the hypothesis on the lipid-lowering potential of melatonin and its antiatherogenic action.
